# Extracellular Vesicles: Novel Potential Therapeutic Agents in Inflammatory Bowel Diseases

**DOI:** 10.3390/cells13010090

**Published:** 2023-12-31

**Authors:** Irene Mignini, Giulia Piccirilli, Fabrizio Termite, Mattia Paratore, Giorgio Esposto, Lucrezia Laterza, Franco Scaldaferri, Maria Elena Ainora, Antonio Gasbarrini, Maria Assunta Zocco

**Affiliations:** CEMAD Digestive Diseases Center, Fondazione Policlinico Universitario “A. Gemelli” IRCCS, Università Cattolica del Sacro Cuore, Largo A. Gemelli 8, 00168 Rome, Italy; irene.mignini@guest.policlinicogemelli.it (I.M.); giulia.piccirilli01@icatt.it (G.P.); fabrizio.termite@libero.it (F.T.); mattia.paratore01@gmail.com (M.P.); giorgio.esposto2@gmail.com (G.E.); lucrezia.laterza@policlinicogemelli.it (L.L.); franco.scaldaferri@policlinicogemelli.it (F.S.); mariaelena.ainora@policlinicogemelli.it (M.E.A.); antonio.gasbarrini@unicatt.it (A.G.)

**Keywords:** inflammatory bowel diseases, extracellular vesicles, novel therapeutic agents

## Abstract

Patients affected by inflammatory bowel diseases (IBD) can nowadays benefit from a growing number of pharmacological options. However, in moderate-to-severe cases, the therapeutic response is still far from optimal, and treatment changes and optimizations are often required. Thus, researchers in this field are strongly engaged in studies aiming to identify new potential therapeutic targets. Extracellular vesicles (EVs) are tiny subcellular bodies with a phospholipid bilayer envelope containing bioactive molecules, which are released from different cells and are involved in intercellular communication. Recent pre-clinical data show their emerging role in the pathogenesis and treatment of IBD. In our review, we summarize current evidence about the function of EVs as active therapeutic agents in ulcerative colitis and Crohn’s disease, analyzing the properties of EVs derived from different cellular sources and the mechanisms through which they may improve intestinal inflammation.

## 1. Introduction

Inflammatory bowel diseases (IBD), including the two major entities of Crohn’s disease (CD) and Ulcerative Colitis (UC), cause chronic inflammation of the gastrointestinal (GI) tract, which may be associated with a wide range of extraintestinal manifestations and present with a largely heterogeneous disease course, from mild forms to serious and potentially lethal complications. In the most severe cases, symptoms may be debilitating with a detrimental impact on patients’ quality of life [1,2] and a not negligible economic burden, in terms of direct costs due to health care system utilization and indirect costs due to the loss of productivity for both affected individuals and caregivers [3].

IBD pathogenetic mechanisms are complex and not completely elucidated yet. It is widely recognized that IBD pathogenesis is multifactorial and includes genetic, environmental, and microbial factors. Specific monogenic mutations can be responsible for IBD occurrence, especially in early-onset manifestations [4], but genetic factors also contribute to non-monogenic IBD, explaining why the risk of developing UC or CD is much higher in cases of affected family members [5]. However, genetics alone does not represent the only cause of IBD. Environmental factors, notably smoking, a high-fat diet, and obesity, strongly enhance gut inflammation [6]. More recently, the influence of gut microbiota has emerged due to its crucial role in maintaining intestinal homeostasis and gut barrier function and its heterogeneous interconnections with dietary elements, epithelial cells, and the immune system [7,8]. Gut barrier integrity is essential to preventing gut inflammation, and a dysregulated barrier leads to an altered epithelium, increased permeability, and bacterial translocation, resulting in immune system activation [9].

Multiple molecular pathways involving both innate and adaptive immunity have been investigated to identify targets for pharmacological treatments. Thus, the therapeutic armamentarium has widely expanded in the last decades, and nowadays, it encompasses not only conventional drugs such as corticosteroids, aminosalicylates, and immunosuppressors, but also a rapidly increasing number of biologics and small molecules. In cases of moderate or severe disease, IBD physicians can rely on anti-tumor necrosis factor alfa (anti-TNFα), anti-integrins, anti-interleukin 23 (anti-IL23), and Janus kinase inhibitors (anti-JAK) [10,11]. New drugs, such as anti-sphingosine 1 phosphate (anti-S1P), have more recently appeared in the IBD landscape, and several clinical trials are currently testing different molecules [12].

Unfortunately, although such drugs have undoubtedly revolutionized the IBD disease course, their efficacy is still far from being optimal, and many patients often relapse and need treatment optimization. According to a recent network meta-analysis about patients with moderate-to-severe UC, after the induction period, clinical response varies from 40% for adalimumab to 77% for upadacitinib, while clinical remission ranges from 17% for golimumab to 50% for upadacitinib [13]. Similar results have been achieved in the pharmacological response in CD [14,15,16]. Thus, a considerable percentage of subjects still experience primary or secondary failure in the currently available treatments.

Therefore, it is crucial to keep investigating new potential therapeutic targets to offer other options to IBD patients. In such a context, an intriguing topic has been raising researchers’ interest in recent years: the role of extracellular vesicles (EVs), tiny vesicles secreted by most eukaryotic and prokaryotic cells, which contain various components, that might open promising perspectives for IBD treatment. As is well illustrated in a recent review by Chen et al., EVs are deeply involved in IBD pathogenesis and may represent useful diagnostic and therapeutic tools. In particular, they may play a double pharmacological role as active agents, due to the beneficial properties of their cargoes and surface proteins, or as carriers for drug delivery, developed thanks to nanotechnologies that allow the incorporation of therapeutic molecules inside the EVs [17]. The latter application has been summarized by Mori et al., who have provided a comprehensive synopsis of the main points of strength and limitation of this new type of drug carrier in different inflammatory diseases [18].

Instead, in our review, we specifically focus our attention on the role of EVs as active therapeutic agents in UC and CD. We first synthetize the classification, composition, and functions of EVs. Subsequently, we thoroughly analyze the properties of EVs derived from different cellular sources and provide an updated overview of studies that investigate their efficacy in improving GI inflammation in IBD. Data presented in our manuscript refer mainly to pre-clinical studies. Indeed, being that EVs are so innovative a field of research, evidence on human beings is still lacking.

## 2. EVs: Definition, Classification, and Function in the GI Tract

EVs are heterogeneous subcellular bodies that carry bioactive molecules and endocellular plasmatic material enclosed in a phospholipid bilayer envelope containing membrane proteins and glycoproteins [19].

Exosomes, microvesicles (MVs), and apoptotic bodies (ApoVs) are the three main categories of EVs, differentiating based on size and biogenesis. The size range of exosomes is 30–150 nm. Their production starts with plasma membrane endocytosis and continues within the multivesicular endosome, which is finally released into the extracellular environment through exocytosis, thus creating exosomes. MVs are produced by the process of plasma membrane budding and can have sizes ranging from 50 to 1000 nm [20,21]. ApoVs are the biggest class of EVs (size range is 1000–5000 nm) derived from membrane blebbing during apoptotic processes, although recent evidence suggests a subclassification of these molecules [22].

EVs can potentially contain every single molecule present in the cell of origin, including lipids and sphingolipids, proteins, and genetic material such as messenger ribonucleic acid (mRNA), microRNA (miRNA), and non-coding RNA [23]. While the spectrum of these molecules varies depending on the source cells, EVs also contain proteins that are crucial to their cycle and participate in fundamental cellular processes [17].

When released, EVs diffuse across the extracellular milieu and are highly involved in intercellular communication. They are able to travel long distances and influence multiple organ systems through the medium of internalization by the target cell with subsequent release of their contents or by adhesion to the surface and induced signaling [24]. Different membrane proteins activate specific metabolic pathways by acting as ligands for host cell receptors. Otherwise, EVs can fuse with receiving cells and release their contents into the host cell via endocytosis or internalization [25]. Upon discharge, the EV’s contents have the potential to modify the receiving cell’s metabolism and gene expression, influencing physiological functions as well as pathogenic mechanisms [20].

In the GI tract, EVs can have different types of source cells, including immune cells, epithelial cells, and gut microbiota composing bacteria; they can also derive from ingesta, being able to resist digestion and low-pH [26,27]. Regarding their role in the GI tract, EVs participate in the development and maintenance of the GI epithelium, influencing enterocyte migration and proliferation [28], as well as the maintenance of the GI barrier integrity by strengthening cellular connections [29]. Additionally, EVs support the maintenance of immunological tolerance of the gut barrier and the “beneficial” structure of the microbiota by regulating immune system activity and the balance between pro- and anti-inflammatory states. EVs appear to be involved in the toll-like receptor (TLR) pathway-mediated activation of the innate immune system [30], the differentiation of various macrophage phenotypes [31], antigen presentation [32], and the interaction between distinct classes of mucosal-associated lymphocytes [33].

**Figure 1:**
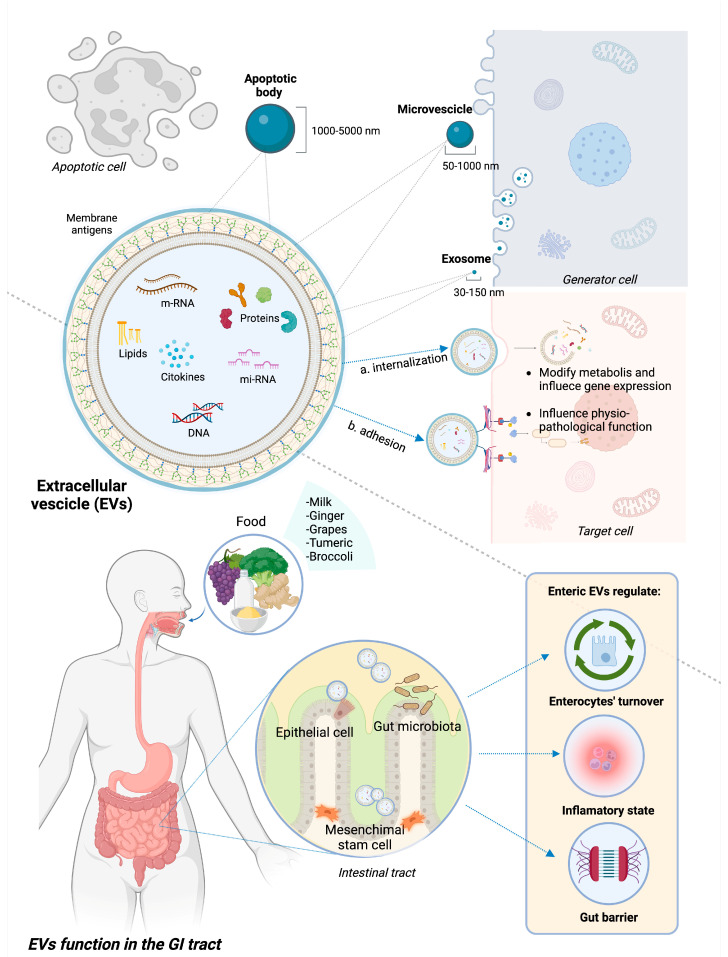
**Structure and functions of EVs in GI tract.** EVs encompass apoptotic bodies, microvesicles, and exosomes, differentiated based on their size. EVs are extracellular bodies with a phospholipid membrane containing heterogeneous molecules, such as proteins, lipids, and genetic material. They interact with target cells through either internalization and subsequent release of the internal content or adhesion to specific receptors and subsequent activation of intracellular pathways. In IBD pre-clinical models, EVs deriving from epithelial cells, mesenchymal stem cells, gut microbiota, and some ingesta proved to have a beneficial role on GI inflammation through different mechanisms, notably modulating pro/anti-inflammatory status, promoting intestinal re-epithelization and strengthening gut barrier.

Figure 1 represents the structure and composition of EVs, underlining the different source cells and therapeutic functions in GI inflammation.

## 3. EVs as Therapeutic Agents in IBD

Since their discovery, EVs have been found to be secreted by numerous cell types in both normal and pathological situations and to be present in different biological fluids. As the progenitor cells, they are used to transfer small molecules to other cells. EVs are easily accessible and can be used for diagnostic, prognostic, and therapeutic applications.

Due to their features, they appear to be useful natural tools to deliver drugs. Thanks to the development of nanotechnologies, it is possible nowadays to incorporate multiple molecules inside EVs, thus improving drug solubility, bioavailability, and selectivity and reducing adverse events [34]. In IBD, EVs carrying different molecules have been tested, including in approved therapies and new drugs. For instance, Wang et al. encapsulated methotrexate in grapefruit-derived nanovesicles to create an oral delivery vehicle to target intestinal macrophages for the treatment of intestinal inflammatory-related diseases. They achieved a powered effect and lower toxicity when compared to free methotrexate [35]. However, nanocarrier EVs’ results are especially promising in delivering nucleic acid therapies, for which oral administration is definitely challenging. Recent evidence shows how different anti-inflammatory RNAs (such as miR-146a and miR-200b) may improve gut inflammation or fibrosis when incorporated into EVs [36,37]. Food-derived EVs are particularly useful for this purpose, considering their stability and ability to survive in harsh environments such as gastric acidity [38].

Moreover, the therapeutic potential of EVs as active agents has also been investigated in IBD pre-clinical models. Indeed, EVs may have a therapeutic effect not only by delivering other molecules but also directly through their own content (proteins, lipids, and RNA). EVs from different sources, notably mesenchymal stem cells, epithelial cells, gut microbiota, and ingesta, have been analyzed. A recent systematic review and meta-analysis by Hou et al. compared a total of 21 studies about the efficacy of EVs in reducing intestinal inflammation. Despite the heterogeneity among studies, they identified a general trend, evidencing that EVs significantly improved (*p* < 0.05) scores such as disease activity index, myeloperoxidase activity, histopathological score, upregulating the expression of anti-inflammatory cytokines, and downregulating the expression of pro-inflammatory mediators in colitis animals [39]. Current evidence reports that EVs released by different types of cells in the GI tract, gut microbiota, and edible plants play an essential role in the pathogenesis and treatment of IBD, as they represent critical actors in intercellular communication, and their cargoes exert an important effect on immune system modulation. Each of the next subsections specifically focuses on EVs from one of the aforementioned cellular types and their therapeutic potential in IBD.

### 3.1. EVs from Adult Mesenchymal Stem Cells

Mesenchymal stem cells (MSCs) represent a subgroup of heterogeneous, non-hematopoietic fibroblast-like cells, coming from a wide range of sources where they can be isolated, such as adipose and perivascular tissue, bone marrow, synovial membrane, the umbilical cord blood, and tissue. MSCs express specific surface markers (such as CD73, CD90, CD44, and CD105) that differentiate them from hematopoietic stem cells [40,41]. They are capable of multilineage differentiation that can be directed to the mesodermal lineage cells (such as adipocytes, osteocytes, and chondrocytes) and ectodermal lineage cells (neuronal and neuroglial cells) [42]. Furthermore, this type of stem cell has demonstrated adherence during growth, self-renewable capability, pluripotency, and immunomodulatory properties [43]. Such qualities make MSCs widely used in cell therapy, tissue engineering, and regenerative medicine.

Considerable evidence demonstrates the immunoregulatory features of MSCs, which may exert an effect on immune cells by direct contact or paracrine pathways, such as through the secretion of EVs [44]. Indeed, it has been shown that EVs released by MSCs express some of their parent’s cell surface markers, including CD73, CD90, and CD105 and several other adhesion molecules (such as CD29, CD44, and CD73), which enable their homing to the injured and inflamed tissues [45,46,47]. The signaling process initiates with the interaction between EVs and the target cell, facilitated by specific surface receptors and membrane molecules. These components allow membrane fusion with target cells [48]. Moreover, EVs have similar immunomodulatory roles as their source cells because they contain many of the same cytoplasmatic proteins and metabolic molecules, including deoxyribonucleic acid (DNA), mRNA, miRNA, signaling lipids, proteins, and soluble factors such as cytokines, chemokines, and growth factors [43]. Basically, they have shown the same therapeutic efficacy and have been recognized as potential candidates for treating autoimmune diseases.

The ability to modulate the immune system is exerted by MSCs on both the adaptive and innate immune systems by soluble factors and direct cell–cell interactions in response to immune cells. They realize these effects by multiple mechanisms: (1) interfering with human B-cells at multiple levels such as proliferation, differentiation to antibody-producing cells, and chemotaxis [49]; (2) suppressing the T cells proliferation and inhibiting their production of inflammatory cytokines [50]; (3) blocking the differentiation of monocytes into dendritic cells (DCs) and impairing their antigen-presenting capacity [51]; (4) reducing the proliferation and cytotoxicity of natural killer cells [52]; (5) facilitating the generation of regulatory T cells. By releasing pro-angiogenic factors and trophic immunomodulators, they attenuate the immune response and inflammation, reduce ischemic injury, and promote tissue repair and regeneration [53,54]. A well-represented example of the tolerogenic potential of MSCs has been reported by Ciccocioppo et al., who demonstrated in an in vitro model the immunomodulant and immunosuppressive effects of MSCs on gliadin-specific T cells, which are known to induce intestinal lesions in celiac disease. In this study, investigators obtained MSCs and gliadin-specific T-cell lines from allogeneic donors and mucosal specimens from celiac patients, respectively. They evaluated the effect of MSCs in terms of proliferative response and interferon (IFN)-γ production upon long-term T-cell lines and the apoptotic rate and cytokine profile of short-term T-cell lines, respectively. The results were surprising, displaying that MSCs can exert an inhibitory effect on proliferation and INF-γ production on long-term T-cell lines and suppress the expansion of short-term T-cell lines by increasing the apoptotic rate. These results suggest a potential novel use of MSC as a cellular therapy [55].

Recently, it has been demonstrated that EVs derived from MSC have a beneficial effect on IBD models. Yang et al. studied rat models with intestinal fibrosis induced by 2,4,6-trinitrobenzenesulfonic acid solution (TNBS) and observed that miR-200b from EVs can suppress colonic fibrosis by inhibiting the progress of epithelial to mesenchymal transition (EMT), which is considered a novel source of fibroblasts in intestinal fibrosis. They found that miR-200b EVs were able to reverse IEC morphology by increasing the expression of E-cadherin and decreasing vimentin and α-smooth muscle actin, eventually regulating colon EMT by targeting ZEB1 and ZEB2, which are fibrosis-related proteins, by reducing the expression of the transcriptional repressor of E-cadherin. Moreover, this study showed how RNA and proteins packed into EVs could function as endogenous forms when in recipient cells; these findings reveal that EVs can be used as a new delivery system with high transport efficiency and stability [37]. These results are of particular interest, considering that intestinal fibrosis is among IBD-related complications and often follows the location and distribution of inflammation, eventually leading to the thickening of intestinal walls and bowel obstruction. By now, there are no effective pharmacological therapies against intestinal fibrosis; the only successful treatments require surgery or endoscopic dilatations [56]. Another example of the beneficial effect of EVs has been reported by Cao et al., who highlighted how EVs secreted by bone marrow mesenchymal stem cells (BMSCs) can improve colitis both in vitro in lipopolysaccharide (LPS)-treated macrophages and in mice models with dextran sulfate sodium (DSS)-induced colitis, by promoting M2-like macrophage polarization [57]. According to their results, the positive action of EVs in repairing tissue damage and, more specifically, in repairing UC damage is linked to the JAK1/STAT1/STAT6 signaling pathway. In the in vivo model, intraperitoneal injection of EVs improved colitis symptoms by decreasing the secretion levels of pro-inflammatory cytokines (Vascular Endothelial Growth Factor—VEGF, IFNγ, TNF-α, and IL-12) and chemokines (CCL-17 and CCL-24), increasing IL-10, and transforming growth factor (TGF)-β levels. These changes are a consequence of a shift from an M1-like to M2-like profile in macrophages, as demonstrated by the decrease in the expression of the M1 marker (CD86) and increase in the M2 marker (CD163) in colon tissues of colitis mice. However, it is important to clarify that the specific signaling pathways implicated are still not fully known and require additional analysis [57]. Furthermore, Heidari et al. reported the immunomodulatory effect of EVs derived from adipose MSCs when used in intraperitoneal injection in a rat model with DSS-induced colitis. This study evaluated the capacity of EVs to down-regulate the levels of pro-inflammatory cytokines, such as IFN-γ, TNF-α, IL-12, and IL-17, and upregulate the levels of anti-inflammatory cytokines, such as TGF-β, IL-4, and IL-10. Cytokine’s profile change induced by EV administration may indicate a shift in immune responses toward the Th2 and a decrease in the Th1 responses. Since a deficiency in IL-10 levels exacerbates DSS-induced colitis, IL-10 supplementation may become an alternative treatment to improve colitis. Indeed, the authors reported that EV administration reduced colon shortening, body weight loss, bleeding, and colon injury, finally improving the degree of inflammation in DSS-induced acute colitis. Such improvement can be mediated by the molecules contained in EVs, such as matrix metalloproteinase-9 (MMP-9) and VEGF, which are involved in tissue repair, and miRNAs such as miR-21 and miR-29, which have antiapoptotic effects [58].

Although MSC-derived exosomes show increasing potential for human disease, there is still a lack of evidence. Further research should focus on their functions and sorting mechanisms [59].

### 3.2. EVs from Human Umbilical Cord Stem Cells

Mesenchymal stem cells (MSCs) can be isolated from various tissues, but the major sources of MSCs for therapeutic use are bone marrow, umbilical cord, and adipose tissues [60]. Umbilical cord mesenchymal stem cells (hucMSCs) possess the same ability as other MSCs to induce tissue regeneration, to maintain general tissue balance, and to release EVs, mediating different effects on targeted cells by both paracrine and cell-to-cell interactions [61]. They can exert suppressive effects on innate immune cells such as DCs, monocytes, and macrophages, and different studies have demonstrated their involvement in the control of colonic inflammation [62]. The potential role of hucMSC-derived exosomes in the treatment of liver and kidney injuries has been previously investigated by Li et al. and Zhou et al. [63,64]. These studies shed new light on the beneficial impact of EVs derived from hucMSCs, which could be explained by the immunomodulatory protein content (such as IL-10 and TGF-β). Recently, Mao et al. have demonstrated the role of these exosomes in alleviating DSS-induced colitis in mice and have explored the underlying mechanisms of action. They reported that indocyanine green-labeled exosomes from hucMSCs homed to the colon, spleen, and liver of mice 12 h post-intravenous injection and were able to relieve the severity of inflammation in terms of the number of blood stools, weight loss, and spleen size. Furthermore, the expression of anti-inflammatory cytokines such as IL-10 was increased in colon tissue of exosome-treated IBD mice compared to non-treated IBD mice, which, conversely, displayed an increased level of pro-inflammatory cytokines, such as TNF-α, IL-1α, and IL-6. They also provided in vitro evidence that hucMSC-EVs can modulate the expression of inducible nitric oxide synthase and IL-7 genes in macrophages [65].

Similarly, Jiang et al. examined the therapeutic effect of hucMSC-exosomes in repairing the mucosal barrier in colitis-induced mice. Their attention focused on TNF-α stimulated gene 6 (TSG-6), a glycoprotein secreted by MSC with anti-inflammatory, tissue-protective, and tissue-repair effects [66,67]. Studies based on animal models, including IBD, have demonstrated that the biological functions of stem cells are mainly mediated by TSG-6 [68,69]. Song et al. evaluated the effects of intraperitoneal injection of hUC-MSC-exosomes in a murine model of colitis induced by TNBS and DSS [69]. The efficacy of treatment was analyzed in terms of survival rate, body weight, colonic length, and disease activity index. The authors demonstrated that hucMSC-exosomes have significant therapeutic effects on both DSS- and TNBS-induced colitis models, affecting the intestinal immune response in terms of stimulation of Th2 cells and reduction of Th17 cells in mesenteric lymph nodes. In particular, treatment with TSG-6 has been associated with a higher survival rate and an improvement of colonic inflammation in treated mice compared to controls. These results underline the role of TSG-6 as a potential novel therapeutic approach for IBD and other autoimmune diseases [70].

### 3.3. EVs from Intestinal Epithelial Cells

The essential function of the intestinal epithelium is to act as a barrier that regulates interactions with luminal contents. It maintains intestinal homeostasis by acting as a basic immune system, regulating the inflammatory response and communicating through its vesicles with pathogens and the immune system. Intestinal epithelial cells (IECs) are capable of releasing EVs from either the apical or basolateral side [33]. As well as the originating cells, IEC vesicles express on their surface immunomodulatory molecules whose role can be associated with B-cell maturation; they are also involved in promoting a tolerogenic evolution of DCs and macrophages by releasing immunoregulatory signals such as TGF-β [71]. Indeed, it has been demonstrated that EVs derived from IECs can induce the expression of TGF-β after internalization in DCs, which in turn enhances T reg cells to drive a tolerogenic response [72]. Although IECs are not professional antigen-presenting cells, they interact with DCs to potentiate antigen-presenting capacity.

Finally, it has been demonstrated that EVs derived from IECs are necessary to develop adequate responses in physiological and pathological states by both innate and adaptive immune cells. Their role of inducing tolerogenic DCs is considered crucial to maintaining intestinal homeostasis. Focusing on IBD, pathogenesis involves a breakdown of innate immunity, as already mentioned, which may be subsequent to the breakdown of intestinal immunotolerance, the capacity of the immune system to display hyporesponsiveness or unresponsiveness to antigens derived from food intake or self-antigens. By now, factors that have been revealed to take part in maintaining gut environment are T regs, DCs, CD8+ T cells, ϒδT cells, regulatory B cells, immunoglobulins A, commensal bacteria and immunosuppressive cytokines, such as TGF-β1 and IL-10, but mechanisms behind the regulation of the immunotolerance are still unclear. In 2016, Jiang et al. demonstrated that immunosuppressive EVs with high levels of TGF-β1 are produced by IECs, and the intravenous injection of these EVs into DSS-induced IBD mice decreases IBD severity by inducing regulatory T cells and immunosuppressive DCs. On the contrary, the inhibition of endogenous EV production promotes IBD development. Moreover, EVs tend to localize in the intestinal tract associated with epithelial cell adhesion molecules (EpCAM). The knockdown of EpCAM expression in vivo worsens murine IBD and compromises the protective effect of EVs from IECs in murine IBD [73]. In addition, in the same study, the authors described an increased level of TGF-β1 and p-extracellular signal-regulated kinase (p-ERK) in intestinal tissues of IBD patients, compared to healthy control people. They suggested that TGF-β1 expression is upregulated in IBD patients, probably in an ERK-dependent manner, underlying the complex role of TGF-β in gut inflammation. The authors hypothesize that TGF-β increase in IBD patients may represent a compensatory mechanism through which IECs start to produce anti-inflammatory mediators to reduce inflammation levels. Globally, the results of this study profile a promising strategy to restore immune balance in the GI tract in IBD by increasing EV release from IECs or activating ERK signaling in IECs [73].

Consequently, it is reasonable to consider IEC-derived EVs as a new potential therapeutic intervention for IBD.

### 3.4. EVs from Intestinal Commensal and Pathogenic Microbes

The process of production and release of vesicles from microbial surfaces is evolutionarily conserved across eukaryotes, bacteria, and archaea [74]. Bacteria-released membrane vesicles (20–400 nm) are regarded as MVs or outer membrane vesicles (OMVs) based on whether they are Gram-negative or Gram-positive [33]. OMVs can transport a variety of biomolecules, such as enzymes, toxins, antigenic determinants, nucleic acids, and metabolites. OMV contents are preserved from enzymatic degradation by a lipid bilayer envelope that protects them against the harsh extracellular environments of the GI tract.

Microbiota homeostasis is the key to maintaining a healthy human state since the disruption of the balance between host and commensal microbes in the intestine has been implicated in the pathogenesis of many diseases, including IBD [75]. OMVs can be secreted from pathogenic and commensal bacteria, resulting in immunomodulation and the corresponding signaling pathways. Also, the EVs derived from physiological fluids can influence the intestinal microbiota. MVs derived from commensal bacteria of the GI tract are distributed all over the gut lumen with a variety of biomolecules, nucleic acids, enzymes, toxins, and metabolites.

Sometimes, microbiota-derived EVs play a negative role in gut homeostasis. An example is the discovery made in 2003 by Ismail et al. about OMVs from Helicobacter pylori (Hp), which can be accepted by host cells and stimulate responses to the bacteria without the need for adhesion of Hp itself to cause damage to the gastric epithelium [76]. OMVs derived from bacteria are covered in ligands of pattern recognition receptors, including DNA, RNA, lipoproteins, LPS, and peptidoglycan, which means they can initiate pro-inflammatory signaling cascades.

On the other hand, OMVs, especially from probiotic bacteria, may help maintain the homeostasis of the GI tract. Intestinal microbes can modulate the integrity of the gut barrier by reinforcing the tight junctions, thus reducing inflammation and other activities [33]. An example of how bacteria can exert both positive and negative effects on the human intestine is represented by *Escherichia coli C25*, which is the first colonized bacterium in the intestine, and its counterpart, *Escherichia coli Nissle 1917* (EcN). The first one can stimulate a mild pro-inflammatory effect on enterocytes by upregulating TLRs in vitro. Probiotic EcN, on the other hand, acts as a good colonizer and plays a beneficial role in the human gut by releasing the protein TcpC to regulate the expression of tight junction proteins in IBD [77].

Interestingly, Wang et al. focused their research on the less explored field of parasites. Previous epidemiologic studies have demonstrated that helminth parasites’ infection is associated with a lower risk of developing autoimmune diseases based on the suppressive activity of helminth-induced regulatory T reg cells or Th2 cells [78]. Assuming the above, we note that helminth parasites could represent a great therapeutic potential for inflammatory diseases, including IBD. In fact, both the protective and therapeutic effects of helminth parasite infection have been confirmed with animal experiments and clinical trials. Thus, intestinal parasites such as *Hymenolepis diminuta*, *Heligmosomoides polygyrus*, and *Trichinella spiralis* have been demonstrated to be able to mitigate dinitrobenzene sulfonic acid-induced colitis in mice [79,80,81]. *Echinococcus granulosus* displayed anti-inflammatory effects in mice with DSS-induced colitis, as well as keeping the integrity of the intestinal mucosa [82], and *Ancylostoma ceylanicum* and *A. caninum* improve pathology in DSS-induced colitis mice by down-regulating the responses of Th1 and Th17 [83]. Wang et al. in their study found that exosomes derived from DCs and treated with *S. japonicum* soluble egg antigens (SEA and SEA-treated DC exosome) were able to attenuate the severity of acute DSS-induced colitis in terms of colon lengths, colon damage, weight loss, stool type, and bleeding, which were improved overall. Treatments with exosomes or SEA were conducted via intraperitoneal injection. These results indicate that SEA-treated DCs are more effective than DC exosomes and may be useful as a new approach to treat IBD [84].

In conclusion, the involvement of immunomodulatory activities in extracellular products derived from commensal bacteria has been noticed in the last fifty years, but the underlying mechanism is not completely clear yet.

### 3.5. Food-Derived EVs

It is well known that dietary habits have an important effect on bowel inflammation, with different dietary patterns having a different impact on symptoms and disease activity [85]. Recently, it has been hypothesized that certain foods may exert their beneficial role by releasing EVs. In contrast to EVs produced by most human cells, food-derived EVs have the advantage of surviving gastric acidity and reaching the bowel, an ability that makes them particularly interesting for therapeutic purposes due to their high availability if administered orally [86,87].

Milk is one of the most studied nutritional sources of EVs that have been isolated from both bovine and human milk. The content of the two types of milk is similar and basically composed of proteins and microRNAs, most of which are involved in intestinal barrier integrity and tight junction signaling. More specifically, the 40 most frequent types of microRNA are implicated in mTOR, PI3K-AKT, and p53 pathways, while the most common proteins contribute to Ras, PI3K-AKT, Rap1, and MAPK signaling. Bovine and human milk consistently seem to exert similar effects on bowel permeability and inflammation [88]. To clarify milk-derived EV functions on the gastrointestinal tract, Tong et al. developed a protocol to isolate EVs from the entire milk and fed healthy mice with milk containing different amounts of EVs. They observed that EVs enhanced intestinal immunity and modulated gut microbiota and its metabolites [89]. Subsequently, the same group aimed to investigate the therapeutic effect of milk EVs on intestinal inflammation. They first reproduced a monolayer cell model of the intestinal epithelium in vitro and exposed it to DSS to promote epithelial injury, demonstrating that milk EVs were able to reduce DSS-induced degradation of tight-junction proteins. They confirmed their results in vivo by developing mouse models of acute and chronic colitis that received bovine milk EVs via oral gavage. An improvement in clinical, histological, and serological markers of disease was observed. Interestingly, to further support these findings, they showed that EV-depleted milk supernatant did not improve intestinal barrier impairment or colonic inflammation [88]. The beneficial effects of EVs seem to follow different pathways, including innate immunity through the inhibition of TLR4 and NLRP3 inflammasome, adaptative immunity through the restoration of the balance between T helper 17 and regulatory T cells, and, finally, gut microbiota [27]. An action of EVs on gene expression has also been described: Mecocci et al. analyzed an in vitro model of intestinal inflammation and noted that, after administration of EVs from bovine milk, there was a down-regulation of most pro-inflammatory genes and cytokines [90].

Another paragraph about potential therapeutic EVs from alimentary sources concerns edible plant-derived EVs (pEVs). Even if the general structure is similar to mammalian-derived EVs, pEVs present some specific characteristics, notably the different phospholipid composition of the external bilayer, which contributes to their resistance to degradation in the upper GI tract [87]. They are particularly enriched in lipids, which are responsible for the induction of a tolerogenic effect and regulation of DC [91]. Moreover, pEVs typically have a lower concentration of protein content, which is generally represented by cytosolic proteins and channels or transporters [92]. The great variety of sources, corresponding to multifaceted mechanisms of action, has stimulated researchers’ interest so that the therapeutic potential of pEVs has been tested in multiple fields, such as digestive, respiratory, neurological, circulatory, endocrine dextran sulfate sodium, genitourinary, and musculoskeletal systems’ diseases [92].

As for IBD, pEVs from different fruits, vegetables, and spices have been tested on mouse models of DSS-induced ulcerative colitis. Thus, it has been observed that exosomes extracted from grapes are especially enriched in phosphatidic acids; lipids from this type of pEV interact with intestinal stem cells, promoting their proliferation and intestinal tissue renewal after injury. Liposome-like nanoparticles assembled with grape-derived lipids are able to target intestinal stem cells, confirming the previous findings [93]. EVs from broccoli proved to improve colitis by reducing the expression of TNF-α, IL-17, and IFN-γ while decreasing IL-10. Moreover, they prevent DC activation and monocyte recruitment [91]. When investigating anti-inflammatory dietary sources, studies have also included curcumin, a well-known spice for its anti-oxidant, anti-microbic, and wound-healing properties, which are extracted from the turmeric plant. Recently, Gao et al. found that oral administration of nanovesicles from fresh turmeric helps restore damaged epithelium by stimulating macrophage transformation from M1 to M2 phenotype and modulating gut microbiota [94]. Interestingly, ginger has proved to have a positive effect not only on gut inflammation but also on colitis-related cancer. Indeed, Zhang et al. conducted a study on colitis and colitis-associated cancer mouse models. According to their results, oral administration of ginger-derived EVs reduced clinical signs of disease activity and serological levels of pro-inflammatory cytokines, promoted intestinal re-epithelization, and decreased the risk of developing colon tumors [95].

The main actions of pEVs from different sources are schematically summarized in Table 1, highlighting the laboratory models (in vitro or in vivo) on which they have been tested.

Despite such promising results, research on the therapeutic role of food-derived EVs in IBD is still at an embryonic stage. As Mu et al. have synthetized in their recent review on the topic, only a few pioneering studies testing pEVs on patients affected by different diseases have been registered on ClinicalTrials.gov. In particular, one phase I trial (NCT04879810) involves IBD patients randomized into three groups to receive ginger or curcumin or ginger plus curcumin, aiming to assess their efficacy on symptom improvement [96]. Results of such and further trials are definitely needed to provide new insights into the role of diet in IBD. Indeed, a deeper understanding of the mechanistic effects of certain foods on gut inflammation may help to develop new therapies and define more evidence-based dietary programs.

### 3.6. EVs-Based Therapy: Future Challenges

EVs appear to be promising therapeutic agents in IBD. However, their application in clinical practice is still encumbered by multiple limitations.

Isolation, purification, storage, and correct characterization of EVs are still complex processes requiring high costs, qualified operators, and standardized techniques. Currently, different isolation methods exist without any commonly accepted protocols, thus affecting data interpretation and comparison. Combining several methods in order to reduce limitations related to a specific technique has been suggested [97]. Furthermore, quantifying the exact number of EVs per unit, as well as the amount of proteins, lipids, and other specific molecules, remains difficult but crucial. Similarly, assessing the right location (surface vs. cytosol) of EV components would be of utmost importance to achieve a better understanding of EV functions. The International Society for Extracellular Vesicles tried to answer these questions through a position statement published in 2018, providing specific technical indications about all the aforementioned aspects [98].

When we consider EV therapies in vivo, another essential issue is represented by the administration route. Indeed, per os administration is largely desirable in BD to enhance patients’ compliance and grant a direct effect of the therapeutic molecule on the affected site. However, after ingestion or oral gavage, most EVs show poor stability in the GI tract, basically due to little survival capacity in the harsh gastric environment. More specifically, as mentioned before, food-derived EVs also proved to be highly stable in low pH conditions, thus raising particular interest not only as active therapeutic agents but also for drug delivery [38,87]. Conversely, MSC or IEC-derived EVs show poor stability in acid environments; current studies on animal models mainly concern intravenous or intraperitoneal injection of EVs, and data comparing different administration modalities are still scarce. To date, achieving satisfying oral bioavailability of EVs from human cells requires complex strategies. To this purpose, some engineered EVs have been created with advanced nanotechnologies aiming to protect them from gastric degradation [99]. Recent results appear promising but need to be confirmed by larger studies on animal models and human beings.

## 4. Conclusions

EVs are multifaceted subcellular bodies that appear particularly intriguing due to their external phospholipid bilayer membrane and heterogeneous internal content. Their specific structure allows them to diffuse across the extracellular space and interact with target cells, making EVs crucial actors of intercellular communication and enabling them to activate different molecular pathways depending on their content and targets. In IBD, EVs from mesenchymal stem cells, intestinal epithelial cells, gut microbiota, pathogen bacteria, and ingesta have been investigated as new potential therapeutic agents. Current data are promising. They describe the role of EVs in modulating the immune system, reducing pro-inflammatory cytokines, decreasing colonic fibrosis, promoting intestinal re-epithelization, and strengthening tight junctions and gut barriers. However, by now, the only available results are based on pre-clinical data, in vitro on cellular models, or in vivo on mice with DSS-induced colitis. Moreover, most of the research focuses on colon inflammation, and models for Crohn’s disease are limited. The application of EVs on human beings represents the next research challenge in this field: to confirm current achievements and propose new therapeutical options for IBD patients.

## Figures and Tables

**Table 1:** Actions of pEVs from different sources as active therapeutic agents in IBD. Both in vitro and in vivo studies are reported.

**IN VITRO MODELS**					
**Source**	**Model**	**Action**		**Reference**	
Ginger	Caco-2 cells	Promoting wound-healing		Zhang et al. (2016) [95]	
Turmeric plant	LPS-treated RAW264.7 macrophages	Decrease in TNFα, IL-6, and MCP-1 Promoting macrophage M2 phenotype		Gao et al. (2022) [94]	
Bovine milk	RAW264.7 macrophages	Inhibition of TLR4 and NLRP3		Tong et al. (2021) [27]	
Bovine milk	Co-culture of IFNγ and LPS-treated Caco-2 and THP-1 cells	Downregulation of pro-inflammatory gene expression Decrease in pro-inflammatory cytokines		Mecocci et al. (2022) [90]	
Human and bovine milk	RAW264.7 macrophages Dendritic cells DSS-treated monolayer epithelial Caco-2 cells	Inhibition of TNFα and IL-6; increase in IL-10 Inhibition of TNFα, IL-6, and CD40 (bovine and human milk); increase in IL-10 (only bovine milk) upregulated expression of tight junction proteins, ZO-1 and Occludin		Tong et al. (2023) [88]	
**IN VIVO MODELS**					
**Source**	**Model**	**Action**	**Administration Route**	**Reference**	
Grape	DSS-induced mouse colitis	Induction of intestinal re-epithelization through promoting stem cell production	Oral	Ju et al. (2013) [93]	
Ginger	DSS-induced mouse colitis IL10−/− spontaneous colitis model Colitis-associated cancer model	Clinical: reduced weight loss Histological, decreased inflammatory cell infiltration, activity scores Reduced pro-inflammatory cytokines, increased IL-10 and IL-22 Increased intestinal epithelial cell proliferation Decreased cancer development	Oral gavage	Zhang et al. (2016) [95]	
Broccoli	DSS-induced mouse colitis adoptive transferring T cells to Rag1^−/−^ mouse colitis model antibody-induced colitis in Rag1^−/−^ mice	Clinical: reduced weight loss, reduced colon shortening Histological, decreased inflammatory cell infiltration, activity scores Serological: reduced TNFα, IFNγ, IL-17, increased IL-10 Inhibition of dendritic cell activation	Oral	Deng et al. (2017) [91]	
Turmeric plant	DSS-induced mouse colitis	Clinical: relief of symptoms Serological: reduced pro-inflammatory cytokines, increased IL-10 Promoting macrophage M2 phenotype Gut microbiota modulation	Oral	Gao et al. (2022) [94]	
Bovine milk	DSS-induced mouse colitis	Clinical: reduced weight loss, reduced colon shortening Histological: reduced fibrosis, epithelial disruption, inflammatory cell infiltration Serological: reduced IL-1β, TNF-α, IL-6, IL-2, IL-22 Gut microbiota modulation	Oral gavage	Tong et al. (2021) [27]	
Bovine milk	DSS-induced mouse colitis	Clinical: reduced weight loss, reduced activity scores, reduced colon shortening Histological: reduced fibrosis, epithelial disruption, inflammatory cell infiltration Serological: reduced TNFα, IL-6, IL-17, IL-1β Gut barrier: upregulation of tight-junction proteins and mucins	Oral	Tong et al. (2023) [88]	

LPS: lipopolysaccharides; DSS: dextran sulfate sodium; TNFα: Tumor Necrosis Factor α; IL: interleukin; MCP-1: Monocyte Chemoattractant Protein 1; TLR: Toll-like-receptor; IFNγ: interferon-gamma.

## Data Availability

No new data were created or analyzed in this study. Data sharing is not applicable to this article.
